# Effects of maternal feeding of clofibrate on hepatic fatty acid metabolism in suckling piglet

**DOI:** 10.1186/s40104-024-01104-6

**Published:** 2024-12-05

**Authors:** Jinan Zhao, Brandon Pike, Feng Wang, Lin Yang, Paige Meisner, Yanling Huang, Jack Odle, Xi Lin

**Affiliations:** 1 Laboratory of Developmental Nutrition, Department of Animal Science, North Carolina State University, Raleigh, NC 27695 USA; 2 Present Address: Sales Department, Zinpro Corporation, Eden Prairie, MN 55344 USA; 3 Present Address: Extension Agent, NC State University, N. C. Cooperative Extension-Northampton County Center, 9495 NC Highway 305, PO Box 636, Jackson, NC 27845 USA; 4 Present Address: Key Laboratory of Animal Science of State Ethnic Affairs Commission, Southwest Minzu University, Chengdu, 610041 People’s Republic of China

**Keywords:** Clofibrate, Hepatic fatty acid oxidation and metabolism, Newborn pigs, Postnatal age, PPARα

## Abstract

**Background:**

Energy deficiency is a leading cause of the high pre-weaning mortality of neonatal piglets in the swine industry. Thus, optimal energy metabolism is of crucial importance for improving the survivability of neonatal piglets. The effective utilization of milk fat as primary energy is indispensably required.

**Methods:**

Pregnant sows (*n* = 27) were randomly assigned into 3 treatments. Each treatment received a standard diet (3,265 kcal ME/kg) supplemented with either 0, 0.25% or 0.5% clofibrate (w/w) from d 107 of gestation to d 7 of lactation. The effects of maternal clofibrate on their milk fatty acid (FA) and performance of the piglets were evaluated. The evaluations were performed via measuring sow productive performance, milk FA composition, and hepatic FA oxidation of the piglets at birth and d 1, 7, 14 and 19 after birth.

**Results:**

Maternal supplementation of clofibrate had no effect on reproductive performance of the sows at farrowing and weaning (*P* > 0.05). However, the mortality at weaning was reduced for piglets from sows with 0.25% of clofibrate, and the average weekly (and daily) gain was higher in piglets from sows that received clofibrate than sows without clofibrate in the first week (*P* < 0.0001). Maternal clofibrate increased percentage of milk C12:0 and C14:0 FAs but decreased C18:2 and n-6 polyunsaturated FAs. Maternal clofibrate also increased plasma ketone body levels and hepatic FA oxidation measured at the first day of birth, but the increase was not detected in piglets on d 7, 14 or 19. Clofibrate was not detected in milk collected from the clofibrate-treated sows. The percentage of FA oxidation decreased, and the percentage of FA esterification increased with increasing in postnatal age. Supplemental carnitine increased FA oxidation regardless of succinate dehydrogenase inhibition, and the increase had no effect on FA esterification.

**Conclusions:**

Maternal supplementation of clofibrate during late gestation and early lactation increases hepatic FA oxidative metabolism at birth and improves growth performance of newborn piglets. Maternal clofibrate transfer to suckling piglets via milk was not detected. Carnitine availability is critical for piglets to maintain a high FA oxidation rate during the suckling period.

**Supplementary Information:**

The online version contains supplementary material available at 10.1186/s40104-024-01104-6.

## Background

High pre-weaning mortality of newborn piglets has remained a major economic and animal welfare problem in the swine industry worldwide for decades. The problem is associated with the decreased nutrient efficiency and growth rates, and increased treatment costs. Mortality has been reported recently to average 13% in several major western developed countries ranging from 5% to 35% [1] and is even higher in some countries [2]. The estimated economic loss is enormous for the swine industries.

Pre-weaning mortality occurs primarily in the first week (especially first 2–3 d) after birth, accounting for 50% of the mortality. The most common explanations for the high mortality rate are starvation and diarrhea (about 69%), although it could be the consequence of a set of complex interactions between the sow, the piglet, and the environment. Pre-weaning mortality due to starvation and diarrhea is highly associated with the underdevelopment and dysfunction of the liver and gastrointestinal tract of the newborn piglet. Evidence shows that weak and malnourished newborn pigs have a greater risk of being exhausted, infected, and crushed during the suckling period, especially in the first week. Because of the limited energy reserves and the lack of active immunity at birth, efficient intestinal uptake, and effective hepatic metabolic use of colostrum (milk) are extremely important for both supplying nutrients (energy) and supporting development of immune system. Thus, early nutritional and immunological interventions have been imperative for improving the survivability of neonatal pigs.

The energy reserve of newborn piglets is only 1%–2% body fat at birth, which is approximately half of that present in lambs and calves [3, 4]. This special energy status puts them at a disadvantage in the hypothermic environment for colostrum/energy uptake, and the energy source abruptly alters from primarily carbohydrate in utero to predominantly fat in milk after birth, suggesting that effective uptake and utilization of fat (energy) is indispensably required. Unfortunately, newborn piglets have a low capacity of oxidizing fat and generating ketone bodies as well as a higher fatty acid (FA) esterification rate [5–8] compared with neonates from other species, indicating that newborn piglets cannot expeditiously use milk fat for their energy requirement. To improve the efficiency of energy utilization, we have focused on the role of activating peroxisome proliferator-activated receptor-alpha (PPARα) in oxidation and regulation of hepatic FA oxidation. Feeding clofibrate, a pharmaceutical PPARα agonist, to pregnant sows [9] or directly to newborn piglets [10] increased the gene expression of the key enzymes associated with the FA oxidation in liver. The up-regulation of gene expression significantly increased FA oxidation in both mitochondria and peroxisomes during development, and the increased FA oxidative capability tended to improve the growth rate of suckling piglets*.*

This study was designed to evaluate the effect of maternal activation of PPARα on milk FA composition and utilization as well as growth performance of newborn piglets during the neonatal suckling period. Clofibrate, as the PPARα activator, in the sow diet can be absorbed and transferred to fetal pigs via placental transfer, inducing hepatic FA oxidation in newborn piglets by increasing the related enzyme activities of the piglets [9]. We hypothesized that maternal supplementation of clofibrate during late gestation and early lactation could maintain activation of PPARα, increase energy utilization and improve growth performance in piglets during the suckling period. To evaluate our hypothesis, the effects of clofibrate with the increased postnatal age on milk FA composition, growth performance, and haptic FA metabolism were examined throughout the suckling period.

## Materials and methods

### Animals and experimental procedures

Twenty-seven pregnant sows were randomly assigned to three groups of 9 sows each group based on parity and body weight. The sows were housed individually and received a standard commercial gestation diet and lactation diet (3,265 kcal ME/kg) containing either 0, 0.25% or 0.5% clofibrate (w/w) from d 107 of gestation until d 7 of lactation based on our previous studies [9, 11]. The sows were monitored on the predicted delivery date. After farrowing, blood samples were collected and the total number of pigs born, number born alive and birth weight were recorded. The litter size then was adjusted to even as possible within 24 h via cross fostering in the treatment group. Milk samples were collected on d 1, 3, 5, 7, 10, 14 and 19. The body weight of piglets was recorded individually on d 1, 7, 14 and 19. The mortality and health status were recorded daily during the experiment. A piglet with average body weight was removed from each litter and euthanized by American Veterinary Medical Association (AVMA)-approved exsanguination after anesthetization at d 1, 7, 14 and 19. Liver samples were collected for enzymatic and molecular determinations as described below. Tissues collected for clofibrate, enzyme and gene expression assays were frozen in liquid N_2_ immediately and stored in −80 °C until analysis. The animal care and all experimental procedures were approved by the Institutional Animal Care and Use Committee (IACUC) at North Carolina State University, IACUC ID 16-142.

### Fatty acid metabolic measurement

FA oxidation and esterification in liver were measured in whole fresh tissue homogenate using ^14^C labeled-oleic acid (1 mmol/L; 0.25 μCi/μmol). Measurements were conducted in the absence and presence of carnitine (1 mmol/L) pre-incubated with or without malonate (10 mmol/L), an inhibitor of succinate dehydrogenase. ^14^CO_2_, ^14^C-labeled acid soluble products (ASP) and ^14^C-labeled esterified FA products (ESP) produced from the oleic acid oxidation in the tissue culture were determined using scintillation counter, and metabolic (esterification and oxidation) rates were calculated based on the measured CO_2_, ASP and ESP following the procedures as described by Odle et al. [12].

### Biochemical analysis

Clofibrate concentration in plasma, milk and tissues were examined using HPLC methods established in our laboratory [9]. Milk FA profile was analyzed using GC/MS methods as described by Lin et al. [13]. Plasma total ketone bodies were measured in a plate reader (BioTek Instruments, Inc.; Winooski, VT 05404, USA) following the procedure described by Kientsch-Engel et al. [14], and acetate was analyzed using a kit purchased from Sigma-BioVision (Milpitas, CA 95035, USA) following the kit instructions. Protein in tissue homogenates was measured using plate reader following the biuret method [15].

### Gene expression

*PPARα* and its target genes related to FA oxidation and ketogenesis such as carnitine palmitoyltransferase I and II (*CPT I* and *II*), acyl-CoA oxidase (*ACO*), 3-hydroxy-3-methylglutaryl-CoA synthase (*HMGCS*), acetyl-coenzyme A acetyltransferases *(ACAT*) were measured by qPCR as described previously [16, 17]. Primer sequences are reported in Additional file 1. Relative changes in gene expression were calculated from the real-time RT-PCR data using the 2^−ΔΔCT^ method [18].

**Table 1:** The effect of dietary supplementation of clofibrate on sow performance

Item	Body weight, kg			Parity	Weight loss, kg			Litter size, n			Litter birth weight, kg	
	Starting	Farrowing*	Weaning		Farrowing*	Weaning*	Total	Live	Mummy	Stillborn	Total	Average^#^
Con	240.5	229.4	232.6	2.3	−9.88	+0.21	−7.86	12.8	0.56	1.11	17.7	1.45
0.25% Clof	239.2	242.1	239.8	2.4	−2.09	+4.94	+0.60	9.1	0.67	0.56	14.6	1.62
0.5% Clof	245.1	245.4	230.6	2.4	−13.31	−6.45	−14.56	12.4	0.67	1.11	18.0	1.47
SEM	9.92	9.32	8.39	0.88	5.18	5.02	6.00	1.14	0.45	0.54	1.56	009
*P*-value	0.91	0.45	0.72	0.99	0.32	0.33	0.23	0.07	0.98	0.77	0.26	0.35

*Con *No clofibrate administration, *Clof *Clofibrate. Data are presented as least squares means

*The means were calculated from 8 sows for Con and 0.25% Clof treatment and 7 sows for 0.5% Clof treatment

^#^Average body weight (kg)/live piglet

### Statistical analysis

Data from growth performance and biological assays was subjected to a SAS generalized linear model (GLM) following a randomized complete block (by litter) design with a 3 (clofibrate level) × 4 (lactation day/postnatal age) factorial arrangement. Data from FA metabolism was analyzed using GLM procedure following a randomized complete block design with a split-plot arrangement. The main plot was the animal treatment (maternal clofibrate level × offspring postnatal age), and the subplot was the treatment in-vitro on liver tissue including carnitine and malonate. The least squares means (LSMs) statement was used to calculate the estimated effects and Tukey test was used for the multiple comparisons of the LSMs. Polynomial orthogonal contrasts were performed to determine the linear and quadratic responses to maternal dietary clofibrate level and lactation day/postnatal age of the piglets. To explain the quadratic response better, the optimal model was assessed and reported employing SAS nonlinear regulation procedure. Animal replication for the experiment was projected from power tests using data from our previous studies [19]. Significant differences were declared at a *P* value of ≤ 0.05 and trends were declared when *P* values were 0.05 < *P* < 0.1.

## Results

### Animal performance

#### Sow performance

No differences were detected on sow performance on body weight, litter weight, parity, and percentage of live, mummy and stillborn among the sows received 0, 0.25% and 0.5% of clofibrate in feed (Table 1). However, the number of the live piglets tended to be lower from sows receiving 0.25% clofibrate than 0 and 0.5% of clofibrate (*P* = 0.07).

#### Piglet performance

Maternal clofibrate supplementation had significant impacts on body weight, weight gain and average daily gain of offspring (Table 2) in the first week after birth. The body weight measured was higher in pigs from sows receiving 0.25% clofibrate than pigs from control and 0.5% clofibrate (*P* < 0.001) at d 1, 7, 14 and 19, while the body weight measured in pigs from sows with 0.5% clofibrate was only higher than that from control sows at d 7. No difference was detected between pigs from sows receiving 0.5% clofibrate and control sows at d 1, 14 and 19. The weight gain and average daily gain were also higher in pigs from sows receiving clofibrate than control sows, and from the 0.25% clofibrate than 0.5% clofibrate at d 7 (*P* < 0.0001). No difference was detected among the treatments after 7 d (*P* > 0.05). Overall, the average body weight gain at weaning tended to be higher in pigs from sows receiving 0.25% versus control sows and sows fed 0.5% clofibrate (*P* = 0.07).

**Table 2:** The effect of maternal supplementation of clofibrate on piglet performance during postnatal period

Item	**Mortality, %**	**Body weight, kg**				**Weekly gain, kg**				**Average daily gain, kg/d**			
		D1	D7	D14	D19	W1	W2	W3	End	W1	W2	W3	End
Con	28.9^b^	1.45^a^	2.63^a^	4.50^a^	6.09^a^	1.17^a^	1.92	1.56	4.65	0.168^a^	0.274	0.223	0.245
0.25% Clof	12.4^a^	1.68^b^	3.20^c^	5.23^b^	6.87^b^	1.52^c^	2.04	1.59	5.17	0.217^c^	0.293	0.226	0.272
0.5% Clof	22.5^b^	1.51^a^	2.84^b^	4.77^a^	6.29^a^	1.34^b^	1.92	1.48	4.79	0.197^b^	0.274	0.211	0.252
SEM	4.42	0.034	0.072	0.132	0.179	0.052	0.061	0.055	0.156	0.007	0.008	0.008	0.008
*P-*value	0.045	0.0001	0.0001	0.0008	0.008	0.0001	0.290	0.364	0.071	0.0001	0.290	0.364	0.071

*Con* No clofibrate administration, *Clof *Clofibrate. D = days, W = week and End = at end of the experiment. Date are presented as least squares means

^a−c^The means within a column with lacking a common superscript differ (*P* < 0.05)

### Fatty acid composition in milk

#### The effect of clofibrate on fatty acid composition

Supplemental 0.5% of clofibrate in sow diet during late gestation and early lactation increased milk saturated FAs (SFA) % of C12:0, C14:0, C22:0, monounsaturated FAs (MUFA) C20:1, C22:1, and polyunsaturated FA (PUFA) C20:2 and C20:3 (*P* < 0.05) but decreased % of C18:2 (*P* < 0.05; Table 3). No significant differences were detected between the treatments for all other FAs % (*P* > 0.05). The dietary 0.5% clofibrate also decreased % of total n-6 FA and PUFAs (*P* < 0.05), resulting in an increase in ratio of n-3/n-6 FAs (Table 4).

**Table 3:** The effect of maternal clofibrate on milk fatty acid composition, % (w/w) of total identified fatty acids

Item	**Con**	**0.25% Clof**	**0.5% Clof**	**SEM**	***P*-value**
Saturated fatty acid (SFA)					
C10:0	0.011	0.036	0.059	0.021	0.314
12:0	0.029^a^	0.046^ab^	0.086^b^	0.015	0.046
C14:0	1.588^a^	1.784^b^	1.769^b^	0.058	0.034
C15:0	0.024	0.027	0.076	0.019	0.128
C16:0	30.06	30.97	31.09	0.626	0.439
C17:0	0.482	0.529	0.589	0.071	0.582
C18:0	3.912	3.812	3.890	0.100	0.758
C20:0	0.039	0.037	0.035	0.005	0.843
C22:0	0.023^a^	0.017^a^	0.038^b^	0.005	0.024
C23:0	0.003	0.027	0.002	0.013	0.321
C24:0	0.028	0.030	0.029	0.003	0.839
Monounsaturated fatty acid (MUFA*)*					
C14:1	0.071	0.073	0.115	0.032	0.604
C16:1	10.01	9.764	9.418	0.493	0.712
C17:1	0.080	0.096	0.080	0.010	0.425
C18:1	35.92	35.86	35.55	0.607	0.911
C20:1	0.082^a^	0.086^a^	0.119^b^	0.010	0.038
C22:1	0.012^a^	0.024^a^	0.062^b^	0.008	0.0005
C24:1	0.022	0.021	0.039	0.009	0.259
Polyunsaturated fatty acid (PUFA)					
C18:2	17.94^b^	16.75^a^	16.22^a^	0.406	0.011
C18:3n6	0.139^ab^	0.131^a^	0.189^b^	0.015	0.019
C18:3n3	0.334	0.297	0.321	0.019	0.346
C20:2	0.121^a^	0.122^a^	0.160^b^	0.012	0.057
C20:3n6	0.045^a^	0.044^a^	0.084^b^	0.011	0.016
C20:3n3	0.038	0.014	0.016	0.010	0.155
C20:4n6	0.334	0.325	0.361	0.016	0.298
C20:5n3	0.008^a^	0.003^a^	0.062^b^	0.013	0.010
C22:2	0.007	0.009	0.007	0.002	0.705
C22:5n3	0.034	0.041	0.056	0.014	0.593
C22:6n3	0.024	0.022	0.028	0.002	0.203

*Con* No clofibrate administration, *Clof *Clofibrate. Data are presented as least squares means

^a,b^The means within a row with lacking a common superscript differ (*P* < 0.05)

**Table 4:** The effects of maternal clofibrate on milk total saturated and unsaturated fatty acid composition, % (w/w) of total identified fatty acid

Item	**Con**	**0.25% Colf**	**0.5% Clof**	**SEM**	***P*-value**
Total SFA	34.55	35.53	35.89	0.663	0.334
Total MUFA	46.24	45.92	45.39	0.606	0.623
Total PUFA	19.06^b^	17.72^a^	17.47^a^	0.423	0.018
Total n3 FA	0.436	0.372	0.483	0.036	0.094
Total n6 FA	18.44^b^	17.22^a^	16.82^a^	0.415	0.018
Ratio of n3/n6 FA	0.023^a^	0.022^a^	0.030^b^	0.002	0.043

*Con *No clofibrate administration, *Clof *Clofibrate. Data are presented as least squares means

^a,b^The means within a row with lacking a common superscript differ (*P* < 0.05)

#### The effect of lactation days on fatty acid composition

Lactation days had great impacts on % of FA composition (Table 5). In general, the % of SFA increased with lactation days (*P* < 0.01), except for C10:0, C20:0, C23:0 and C24:0 with a low-level concentration. The % of C16:1 also increased after 1 week, but the C18:1 decreased greatly with the lactation days (*P* < 0.0001). In addition, the % of C20:1 and C22:1 was greater in the milk from d 14 than all other days (*P* < 0.005). The PUFA such as C18:2 increased (*P* < 0.0001), while the % of C18:3n6, C18:3n3, C20:3n6, C20:3n3, C20:4n6, C22:2 and C22:6n3 decreased with lactation days (*P* < 0.05). Thus, the % of total SFA increased (*P* < 0.0001) and the % of total MUFA and PUFA decreased (*P* < 0.0001) with lactation days (Table 6). Significant quadratic changes (*P* < 0.05) were observed for C14:0, C16:0, C18:0, C16:1, C18:1 and total SFA and PUFA (Additional file 2).

**Table 5:** The effect of lactation days on milk fatty acid composition, % (w/w) of total identified fatty acids

Item	**Lactation days**							**SEM**	***P*-value**
	**1**	**3**	**5**	**7**	**10**	**14**	**19**		
Saturated fatty acid (SFA)									
C10:0	0.000	0.002	0.007	0.016	0.089	0.109	0.022	0.032	0.106
C12:0	0.002^a^	0.010^a^	0.028^ab^	0.039^ab^	0.075^bc^	0.138^bc^	0.086^c^	0.021	0.003
C14:0	0.674^a^	1.330^b^	1.486^b^	1.880^c^	2.351^e^	2.090^ cd^	2.186^de^	0.093	0.0001
C15:0	0.031^a^	0.007^a^	0.017^a^	0.012^a^	0.048^a^	0.166^b^	0.016^a^	0.029	0.008*
C16:0	20.41^a^	28.24^b^	30.17^b^	32.63^c^	34.41^c^	34.47^c^	34.63^c^	0.953	0.0001
C17:0	0.080^a^	0.523^b^	0.764^b^	1.067^c^	1.075^c^	0.158^a^	0.066^a^	0.108	0.0001
C18:0	3.524^a^	3.917^ab^	3.773^a^	3.961^ab^	4.500^b^	3.896^ab^	3.531^a^	0.160	0.0002
C20:0	0.033	0.039	0.032	0.036	0.044	0.034	0.042	0.009	0.927
C22:0	0.029^a^	0.011^a^	0.022^a^	0.016^a^	0.025^a^	0.062^b^	0.021^a^	0.008	0.0028*
C23:0	0.003	0.012	0.002	0.001	0.00	0.007	0.049	0.020	0.636
C24:0	0.032	0.027	0.023	0.027	0.039	0.025	0.028	0.004	0.182
Monounsaturated fatty acid (MUFA)									
C14:1	0.073	0.023	0.042	0.131	0.077	0.143	0.119	0.045	0.536
C16:1	5.611^a^	8.412^b^	11.55^bc^	10.65^c^	10.32^c^	10.64^c^	10.93^c^	0.513	0.0001
C17:1	0.077	0.057	0.076	0.071	0.110	0.110	0.099	0.016	0.089
C18:1	41.56^d^	39.15^c^	37.74^c^	34.86^b^	33.48^a^	32.29^a^	32.36^a^	0.994	0.0001
C20:1	0.104^a^	0.070^a^	0.082^a^	0.075^a^	0.079^a^	0.166^b^	0.093^a^	0.016	0.002*
C22:1	0.018^a^	0.010^a^	0.014^a^	0.011^a^	0.020^a^	0.126^b^	0.030^a^	0.012	0.0001*
C24:1	0.030	0.053	0.022	0.021	0.022	0.023	0.019	0.011	0.427
Polyunsaturated fatty acid (PUFA)									
C18:2	26.22^a^	17.53^b^	16.76^b^	14.73^c^	14.51^c^	14.07^c^	14.95^c^	0.640	0.0001
C18:3n6	0.241^b^	0.235^b^	0.123^a^	0.113^a^	0.082^a^	0.193^b^	0.085^a^	0.023	0.0001*
C18:3n3	0.495^c^	0.271^ab^	0.265^ab^	0.254^a^	0.309^ab^	0.351^ab^	0.277^ab^	0.029	0.0001*
C20:2	0.191^b^	0.116^a^	0.111^a^	0.101^a^	0.115^a^	0.199^b^	0.109^a^	0.019	0.0006*
C20:3n6	0.093^c^	0.081^bc^	0.043^abc^	0.029^a^	0.034^a^	0.082^bc^	0.039^ab^	0.016	0.015
C20:3n3	0.084^b^	0.008^a^	0.015^a^	0.015^a^	0.008^a^	0.016^a^	0.011^a^	0.015	0.012
C20:4n6	0.544^e^	0.376^d^	0.353^ cd^	0.294^bc^	0.347^c^	0.244^ab^	0.219^a^	0.027	0.0001
C20:5n3	0.020^a^	0.002^a^	0.004^a^	0.004^a^	0.002^a^	0.135^b^	0.003^a^	0.021	0.0006*
C22:2	0.013^b^	0.009^ab^	0.003^a^	0.007^ab^	0.004^a^	0.011^ab^	0.005^ab^	0.006	0.227
C22:5n3	0.046	0.082	0.033	0.027	0.028	0.034	0.055	0.022	0.488
C22:6n3	0.041^d^	0.016^a^	0.033^c^	0.026^bc^	0.016^a^	0.022^ab^	0.019^ab^	0.004	0.0001

Data are presented as least squares means. *Interaction was detected between maternal clofibrate and lactation days

^a−e^The means within a row with lacking a common superscript differ (*P* < 0.05)

**Table 6:** The effect of maternal clofibrate on milk saturated and unsaturated fatty acid composition, % (w/w) of total identified fatty acid

**Item**	**Lactation days**							**SEM**	*P-***value**	**Interaction** *P***-value**
	**1**	**3**	**5**	**7**	**10 **	**14**	**19**			
SFA	24.14^a^	32.79^b^	34.84^b^	37.81^c^	40.14^c^	39.07^c^	38.49^c^	1.010	0.0001	0.945
MUFA	47.46^a^	47.77^a^	49.65^a^	45.82^c^	43.10^b^	43.50^b^	43.65^b^	0.924	0.0001	0.850
PUFA	27.98^c^	18.67^b^	17.88^b^	15.54^a^	15.45^a^	15.35^a^	15.74^a^	0.647	0.0001	0.260
FAn3	0.688^b^	0.380^a^	0.347^a^	0.327^a^	0.364^a^	0.557^b^	0.351^a^	0.054	0.0001	0.009*
FAn6	27.09^c^	18.17^b^	17.28^b^	15.10^a^	14.97^a^	14.58^a^	15.28^a^	0.632	0.0001	0.306
FAn3/FAn6	0.025^a^	0.022^a^	0.020^a^	0.021^a^	0.025^a^	0.039^b^	0.023^a^	0.035	0.028	0.007*

*SFA* Saturated fatty acid, *MUFA* Monounsaturated fatty acid, *PUFA* Polyunsaturated fatty acids, *FA* Fatty acid. Data are presented as least squares means

^*^Interaction was detected between maternal clofibrate and lactation days

^a–c^The means within a row with lacking a common superscript differ (*P* < 0.05)

**Fig. 1:**
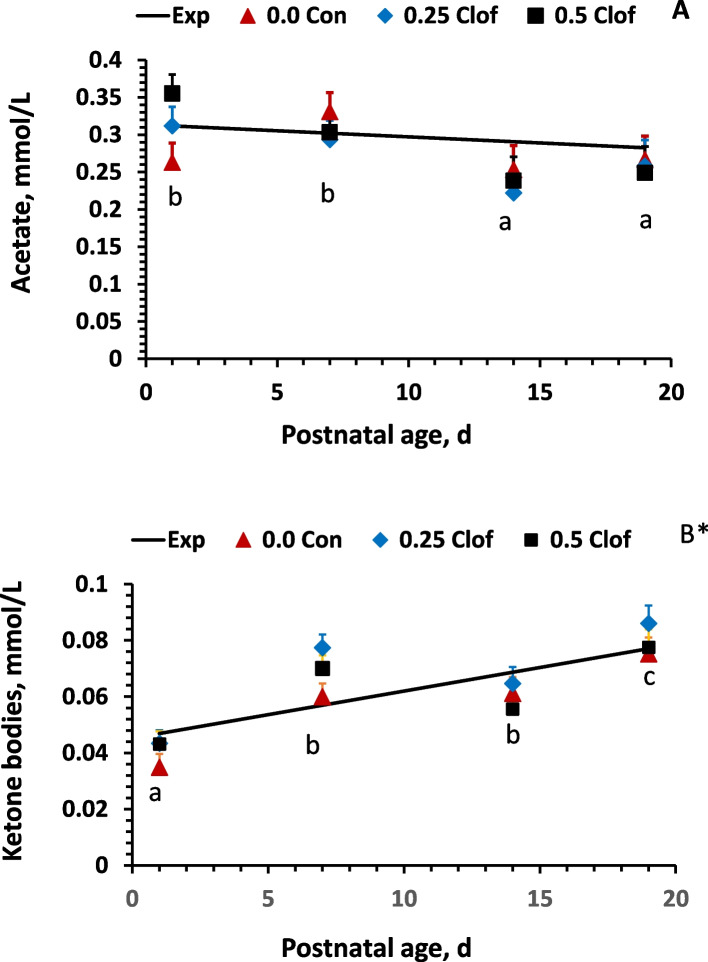
Effect of clofibrate on plasma acetate and ketone bodies during suckling period. Data are least squares means (*n* = 6–9) ± SEM (standard error of the mean); EXP: Expected, Con: Control, Clof: clofibrate. *Indicate significant difference in pigs from clofibrate treated sows compared to control sows (*P* < 0.05). ^a−c^ Denotes significant difference between ages (*P* < 0.05)

Interactions between maternal clofibrate and lactation days were observed for total n-3 FAs (*P* < 0.01), resulting in the ratio of n-3/n-6 FA increasing at 14 d (Table 6).

### Plasma ketone bodies and acetate in piglets

#### The effect of clofibrate on plasma acetate and ketone bodies

Maternal clofibrate supplementation had no impact on the plasma acetate concentration (*P* > 0.05; Fig. 1A). On average, the concentration of plasma acetate was 0.28 μmol/mL. However, the supplementation significantly affected plasma concentration of ketone bodies (*P* < 0.05; Fig. 1B). The concentration of ketone bodies was higher in pigs from sows with 0.25% of clofibrate (0.068 mmol/L) than control (0.058 mmol/L) and 0.5% of clofibrate (0.062 mmol/L).

#### The effect of lactation days on plasma acetate and ketone bodies

Both plasma concentrations of acetate (Fig. 1 A) and ketone bodies (Fig. 1B) in the pigs were affected by the postnatal age (*P* < 0.01). The plasma concentration of acetate decreased in pigs with the increase in postnatal age, while plasma concentration of ketone bodies increased in pigs with the increase in postnatal age. The concentration of acetate (μmol/mL) was on average greater from pigs at d 1 and 7 (0.32) than d 14 and 19 (0.25). The concentration of ketone bodies (μmol/mL) was on average 38% higher from pigs at d 7 and 14 (0.065) than d 1 (0.040), and 24% and 50% higher from pigs at d 19 (0.080) than d 7 and 14. A linear relationship was detected between the postnatal age and plasma acetate (*P* < 0.01) and ketone bodies concentrations (*P* < 0.0001).

No interactions were observed between maternal clofibrate and plasma ketone bodies and acetate in the suckling piglets (*P* > 0.05).

### Hepatic fatty acid metabolism in suckling piglets

#### The effect of maternal clofibrate and postnatal age on oleic acid metabolism

Maternal supplementation of clofibrate increased total hepatic FA oxidation in piglets, but the increase was associated with the supplemental levels of clofibrate and the postnatal ages of piglets (Table 7). Significant interactions were detected between maternal clofibrate levels and piglet postnatal age (*P* < 0.0001). The CO_2_ and ASP increased in piglets at d 1 from sows with dietary clofibrate compared to piglets at d 7, 14 and 19 from sows with clofibrate or piglets at all ages from sows without clofibrate. Moreover, the increase in 1-day-old piglets was greater from sows receiving 0.5% clofibrate than sows receiving 0.25% clofibrate. No difference was detected in all piglets at age of 7 and 14 d cross all treatments, but the ASP was higher in 19-day-old piglets from sows with 0.5% clofibrate than that from control sows and sows with 0.25% clofibrate. The effects of maternal clofibrate and pig postnatal age on total oleic acid oxidation (CO_2_ + ASP) followed the same pattern as for ASP. Furthermore, the oxidative products decreased, and the ESP increased with the postnatal age. The decrease in total oxidative products and increase in ESP were greater in 19-day-old pigs from sows with clofibrate than without clofibrate. No difference was detected in pigs at other ages cross the treatments. A similar pattern was observed for the total metabolic products.

**Table 7:** The effect of maternal clofibrate on hepatic fatty acid metabolism in piglets at different postnatal age

Treatment	**Con**				**0.25% Clof**				**0.5% Clof**				SEM	*P*-value
Postnatal age, d	**1**	**7**	**14**	**19**	**1**	**7**	**14**	**19**	**1**	**7**	**14**	**19**		
Oleic acid metabolism, nmol/h/mg protein														
Oxidation														
CO_2_^*	0.478^a^	0.582^a^	0.703^ab^	0.517^a^	0.835^b^	0.610^ab^	0.466^a^	0.622^ab^	1.147^c^	0.534^a^	0.497^a^	0.657^ab^	0.092	0.0001
ASP^*	9.403^c^	8.478^bc^	9.571^c^	4.550^a^	13.18^d^	9.292^c^	7.968^bc^	6.603^ab^	20.14^e^	9.254^c^	7.398^abc^	7.410^bc^	0.975	0.0001
Total^* oxidation	9.941^c^	9.060^bc^	10.28^c^	5.077^a^	14.01^d^	9.903^c^	8.444^bc^	7.236^ab^	21.28^e^	9.788^c^	7.817^abc^	8.077^bc^	0.928	0.0001
Esterification														
ESP^*	4.438^a^	7.038^abc^	12.83^e^	10.56^cde^	5.391^ab^	8.136^bcd^	10.13^cde^	13.34^e^	10.05^cde^	11.03^de^	8.440^bcd^	19.60^f^	1.437	0.0012
Total metabolite^	14.23^a^	16.22^ab^	23.35^de^	16.54^abc^	19.41^bcd^	18.04^abc^	18.80^abcd^	20.80^bcd^	28.19^f^	20.82^cd^	16.48^abc^	27.91^ef^	1.523	0.0001

*Con* No clofibrate administration, *Clof* Clofibrate. *ASP* Acid soluble products, *ESP* Esterification product. Total oxidation = CO_2_ + ASP and total metabolites = CO_2_ + ASP + ESP. Date are presented as least squares means. ^a−f^The means within a row lacking a common superscript differ (*P* < 0.05). ^A linear response to maternal clofibrate supplemental level was detected (*P* < 0.0001). *A linear response to postnatal age was detected (*P* < 0.0001)

#### The effect of maternal clofibrate and postnatal age on distributions of oleic acid metabolites

No interactions were detected on the distribution of metabolites between CO_2_ and ASP or among CO_2_, ASP, and ESP (*P* > 0.05). Maternal supplementation of clofibrate did not change the distributions between CO_2_ and ASP or among CO_2_, ASP, and ESP (Table 8). However, postnatal age had great impacts on the metabolite distributions among CO_2_, ASP and ESP. Both CO_2_ and ASP decreased with the postnatal age, while ESP increased with the postnatal age. The postnatal age had no effects on the distribution between CO_2_ and ASP, but the percentage of total oxidative products (CO_2_ + ASP) in total metabolites decreased with increasing postnatal age (*P* < 0.05).

**Table 8:** Effects of maternal clofibrate on the distribution of hepatic metabolic products in piglets at different postnatal age

Metabolic product, %	**Treatment**					**Postnatal age, d**					
	**Con**	**0.25% Clof**	**0.5% Clof**	**SEM**	***P*-value**	**1**	**7**	**14**	**19**	**SEM**	***P*-value**
% of oxidation (O)											
CO_2_/O	8.782	8.820	8.040	0.592	0.5690	8.806	7.415	8.009	8.893	0.692	0.3017
ASP/O	91.22	91.98	91.96	0.592	0.5690	91.19	92.59	91.99	91.11	0.692	0.3017
% of metabolism (M)											
CO_2_/M*	3.717	3.631	3.721	0.310	0.9696	5.287^c^	3.845^b^	3.036^ab^	2.590^a^	0.350	0.0001
ASP/M*	45.04	42.63	43.00	2.080	0.2967	60.87^d^	48.40^c^	41.30^b^	23.62^a^	2.418	0.0001
ESP/M*	51.26	53.74	53.28	2.173	0.6876	33.84^a^	47.75^b^	55.66^c^	73.79^d^	2.528	0.0001
O/M*	48.74	46.61	46.72	2.173	0.3063	66.16^d^	52.25^c^	44.34^b^	26.22^a^	2.528	0.0001

*Con* No clofibrate administration, *Clof* Clofibrate, *ASP* Acid soluble products, *ESP* Esterification product. Total oxidation = CO_2_ + ASP and total metabolites = CO_2_ + ASP + ESP. Data are presented as least squares means. ^a−d^The means within a row lacking a common superscript differ (*P* < 0.05). *A linear response to postnatal age was detected (*P* < 0.0001)

#### The effects of carnitine and malonate on oleic acid metabolism

**Fig. 2:**
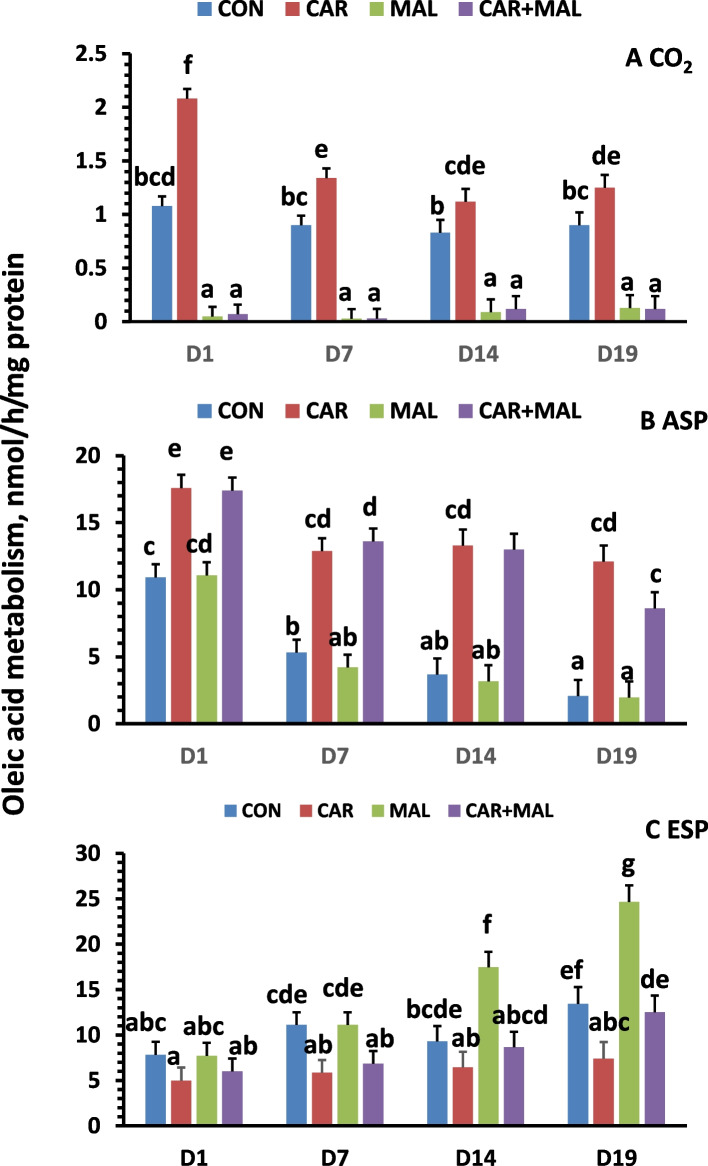
Effects of carnitine and malonate on oleic acid metabolism. CON: Control, CAR: Carnitine, MAL: Malonate, ASP: acid soluble products and ESP: esterification products. Data are least squares means (*n* = 6–9) ± SEM (standard error of the mean); ^a−g^Denotes significant difference between ages (*P* < 0.05)

The interaction between the postnatal age and treatments with carnitine and malonate additions in vitro were detected for oleic acid metabolism and metabolite distribution (*P* < 0.05). Postnatal age had no effect on CO_2_ production (Fig. 2A); whereas ASP (Fig. 2B) decreased, and ESP (Fig. 2C) increased with the postnatal age. The addition of carnitine increased ^14^C accumulation in CO_2_, and ASP across all ages, but the increasing range was affected by age. The increase was much higher for CO_2_ and less for ASP at d 1 than d 7, 14 and 19, and there were no differences among d 7, 14 and 19 (Fig. 2A and B). Adding carnitine had no effect on the ^14^C accumulation in ESP from 1- and 14-day-old piglets but decreased the ESP from 7- and 19-day-old piglets (Fig. 2C). Addition of malonate decreased CO_2_ production but had no effect on ASP as compared with control (*P* > 0.05). However, the ESP was much greater from 14- and 19-day-old pigs than 1- and 7-day-old piglets as well as the control piglets. The addition of malonate and carnitine together had a similar pattern as carnitine only for CO_2_ and ASP but had no effect on ESP with a similar pattern as the control piglets (Fig. 2C).

**Fig. 3:**
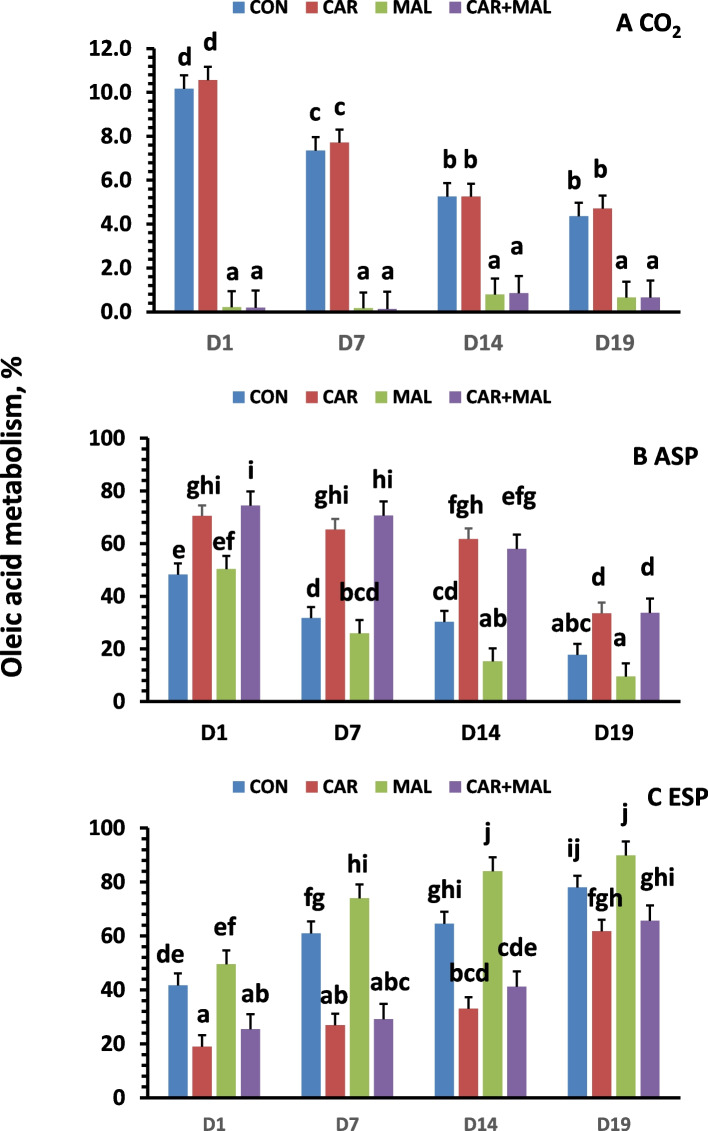
Effects of carnitine and malonate on the distribution of oleic acid metabolites. CON: Control, CAR: Carnitine, MAL: Malonate, ASP: acid soluble products and ESP: esterification products. Data are least squares means (*n* = 6–9) ± SEM (standard error of the mean); ^a−j^Denotes significant difference between ages (*P* < 0.05)

The percentage of CO_2_ (Fig. 3A) and ASP (Fig. 3B) decreased with the postnatal age, while ESP (Fig. 3C) increased. The percentage of CO_2_ followed a similar pattern as the controls after adding carnitine into the culture. However, the percentage of ASP increased, and the percentage of ESP decreased by addition of carnitine. Moreover, the increase in percentage of ASP was greater from 7- and 14-day-old than 1- and 19-day-old pigs, while the decrease in ESP was greater from 7- and 14-day-old than 1- and 19-day-old pigs by addition of carnitine.

### Expression of genes related to fatty acids oxidation and ketogenesis

No interactions were detected between maternal clofibrate supplementations and postnatal age of the pigs for the relative abundance of all measured genes (Table 9) associated with FA oxidation. Clofibrate supplementation increased *PPARα*, *RXRα*, *CAT*,* HMGCS* and *HMGCL* expression, and tended to increase *ACO*,* ACADM* expression, but had no significant impact on *CPTI* and *ACADL* expression. A linear response of gene abundance to clofibrate supplemental concentrations was detected for *PPARα*,* CAT*,* RXRα*,* HMGCS*, and* HMGCL*.

**Table 9:** Effects of maternal clofibrate on genes associated with hepatic metabolic products in piglets at different postnatal age

**Genes**	**Treatment**					**Postnatal age, d**					
	**Con**	**0.25% Clof**	**0.5% Clof**	**SEM**	***P*-value**	**1**	**7**	**14**	**19**	**SEM**	***P*-value**
*CPTIα*	4.57	4.33	5.27	0.97	0.774	1.80^a^	4.45^ab^	7.15^b^	5.51^b^	1.11	0.0078*
*ACOX1*	0.97^a^	1.02^a^	1.47^b^	0.14	0.030*	1.21	1.17	1.15	1.08	0.16	0.950*^
*HMGCS*	15.98^a^	18.64^a^	30.44^b^	3.24	0.006*	8.47^a^	27.51^b^	24.69^b^	26.08^b^	3.76	0.0004*^
*RXRα*	4.51^a^	6.14^ab^	10.85^b^	1.86	0.052*	2.98	7.38	10.70	7.60	2.13	0.0812
*CAT*	2.59	2.51	3.20	0.29	0.205	1.87^a^	2.93^b^	3.17^b^	3.10^b^	0.33	0.0155*
*ABCD3*	1.32	1.23	1.70	0.16	0.084	1.41	1.54	1.13	1.59	0.18	0.3544
*PPARα*	2.46^a^	3.37^ab^	5.56^b^	0.91	0.054*	2.12	4.64	4.59	3.83	1.02	0.2068
*MYLCD*	3.38	2.02	2.58	0.60	0.293	1.11^a^	2.29^ab^	4.18^b^	3.05^b^	0.70	0.0188*
*ACADM*	2.16	1.95	2.56	0.22	0.142	1.70	2.19	1.97	3.03	0.25	0.0040*
*CYP4*	3.34	2.61	3.04	0.424	0.464	1.57	3.44	3.24	3.72	0.482	0.0049*
*ACO*	2.09	2.50	2.92	0.272	0.108*	1.86^a^	2.98^b^	3.00^b^	2.18^ab^	0.312	0.0156^
*CS*	1.37	1.11	1.08	0.106	0.121^	1.18^ab^	1.24^b^	0.86^a^	1.45^ab^	2.418	0.0267^
*ACADL*	2.69	1.70	2.10	0.334	0.122	1.67	249	1.82	2.66	0.385	0.1842
*HMGCL*	2.65^b^	0.80^a^	0.44^a^	0.626	0.032*	0.57	1.10	1.34	2.17	0.717	0.4445
*SLC25a20*	3.13^a^	3.46^a^	4.73^b^	0.327	0.002*	2.54^a^	4.31^b^	4.49^b^	3.77^b^	0.376	0.0007*^

*CPTIα* Carnitine palmitoyltransferase I alpha, *ACOX1* Peroxisomal acyl-CoA oxidase 1 isoform 1, *HMGCS* 3-Hydroxy-3-methylglutaryl-CoA synthase, *RXRα* Retinoid X receptors alpha, *CAT* Catalase, *ABCD3* ATP binding cassette subfamily D member 3, *PPARα* Peroxisome proliferator-activated receptor alpha, *MYLCD* Malonyl-CoA decarboxylase, *ACADM *Acyl-CoA dehydrogenase medium chain, *CPY4* The cytochrome P450 4, *ACO* Acyl-CoA oxidase, *CS* Citrate synthase, *ACADL* Acyl-CoA dehydrogenase long chain, *HMGCL* 3-Hydroxy-3-methylglutaryl-CoA lyase, *SLC25a20* Solute carrier family 25 member 20

*Con* No clofibrate administration, *Clof *Clofibrate. Data (fold) are least squares means. ^a,b^The means within a row lacking a common superscript differ (*P* < 0.05). *A linear response was detected (*P* < 0.05). ^A quadratic response was detected (*P* < 0.05)

Postnatal age had impacts on expressions of *CPTI*,* HMGCS*,* HMGCL*,* ACADM*,* MYLCD*,* ACOX1* as well as* CS *and* CPY4*. A linear response of gene abundance to age was detected for *CPT I*,* CAT, HMGCS, ACADM, MYLCD* as well as *CPY4* (*P* < 0.05). A quadratic response was detected also for *ACO*, *HMGCS*,* RXRα*,* PPARα*,* ACOX1* and *CS* (*P* < 0.05).

## Discussion

Extremely limited information on the effect of maternal feeding of PPARα agonist clofibrate on dam reproductive performance and offspring growth performance is available in the literature. Feeding clofibrate to pregnant C57BL/6 J mice throughout pregnancy had no effect on maternal body weight [20] but feeding pregnant Swiss mice from d 7 to 16 of gestation significantly reduced the fetal mice weight at birth [21]. In our study, administration of clofibrate to pregnant sows in the last week of gestation and the first week of lactation also had no effect on sow’s reproductive performance, which was similar as it observed previously in C57BL/6 J mice. Moreover, no significant difference was detected in the birthweight of newborn piglets from control sows compared to piglets from clofibrate treated sows. The average birthweight of the newborn piglets ranged from 1.45 to 1.67 kg. The lack of impact on birth weight from swine could be due to the different metabolic response to clofibrate administration and the number of sows examined in this study. In rodent species, PPARα is highly expressed in cells that have high FA catabolic rates including the liver, kidney, heart, and skeletal muscle [22], accompanied by a large proliferation of peroxisomes. Unlike rodent species, swine, like humans remains unresponsive to peroxisome proliferation, thus feeding clofibrate in the last week of gestation might not influence seriously on the energy transfer from mother to fetuses. It could be also due to the time of providing clofibrate during pregnancy. One week is only 1/16 of gestation period in swine but 9 d in mice would be more than 1/3 of gestation period. Besides having no impact on birthweight, we found that administrating clofibrate to sows in the last week of gestation increased the average daily gain of piglets during the first week after birth, and the increased average daily gain was not detectable after one week. This finding suggests that maternal clofibrate could stimulate the growth of newborn piglets, but the stimulation has a timeliness depending on the level of clofibrate in the body. Newborn pigs have a limited fat store at birth [23], and sufficient energy available is necessary for supporting their activity and growth after birth. Maternal supplemental clofibrate in the last week of gestation increases energy generation in newborn pigs [9], suggesting that the greater average gain in the first week could be related to the increased in energy generation. Indeed, we found that FA oxidation measured on d 1 after birth was increased 41% and 11.4% in piglets from sows receiving 0.25% and 0.5% clofibrate compared piglets from control sows, in which the total FA oxidation in piglet at birth from sows with 0.5% clofibrate was 1.5 fold of 0.25%, indicating that the placenta transfer was related to the clofibrate dose. In agreement with the increase in hepatic FAs oxidation, the plasma concentrations of total ketone bodies also increased in the piglets from sows receiving clofibrate. However, the promotion on growth was not detectable after d 7, and the stimulation on FA oxidation and ketogenic capacity was not detectable in piglets from sows receiving clofibrate after d 7, which matched the measured growth performance, suggesting that the clofibrate might not be transferable via milk. In support of our data from in-vitro FA oxidation, we did not detect clofibrate or its metabolites in milk and liver tissue samples (data not shown). Similarly, Gessner et al. [24] reported that feeding 2 g of clofibrate/kg diet to lactation sows had no effect on gains of litters.

Milk production, fat content and FA composition are important for postnatal development and growth. Maternal feeding of clofibrate affected milk FA composition, but the effects were limited primarily to the high-level of clofibrate. High clofibrate increased milk medium-chain FA C12:0 and C14:0 concentrations, and unsaturated FA with 20 and 22 carbons with a decrease in C18:2. FA chain-shortening activity was detected previously in outside of mitochondria from rat after feeding clofibrate [25]. Moreover, the increased oxidation of long-chain FA C18:1 and C20:1 induced by clofibrate was associated with an increased capacity of chain shortening in a perfusion study of rat hearts [26]. Evidence from further studies showed that the shortening capacity was associated with the substrate concentration [27], and in vitro clofibrate increased medium-chain acyl-CoA concentration when palmitate was oxidized in liver peroxisomes of rat [28]. Although the increased chain-shortening activity was observed in non-mammary tissues, a similar mechanism could exist in mammary tissue, subsequently affecting the medium-chain FA level in the milk in addition to an indirect effect from liver metabolism. The effect of clofibrate on milk FA composition measured on d 20 of lactation demonstrated that C16:0 increased, and C18:2, C18:3 decreased, suggesting that clofibrate might have an influence on desaturase activity. Similar findings were reported in a previous study, in which the saturated FA increased and the polyunsaturated decreased in milk from treated sows [24]. The influence of clofibrate on desaturase in mammary tissues was not reported, but clofibrate increased hepatic linoleic acid metabolism and biosynthesis of n-6 PUFA in rats [29]. The n-3 and n-6 FA of plant origin can be converted to the C20 and C22 very long chain FAs, in which elongase (palmitoyl-CoA chain elongase) and desaturases (stearoyl-CoA desaturase) play the important regulatory roles [30, 31]. Delta-9, delta-6, and delta-5 desaturase activities in liver of rats were increased by the three fibrates [32].

FA composition can be influenced also by diets and lactation days. However, most of the studies reported previously were focused on the dietary impacts [33–35] but not the kinetic changes in FA composition with lactation days. We found that the concentrations (%) of milk SFA and PUFA changes with the increase in lactation primarily due to the increase in C16:0 and 14:0 and decrease in C18:2n6 and C20:4n6. In addition, the MUFA C16:1 increased and C18:1n9 decreased with the lactation days also following the exponential function (Additional file 2). These findings were similar to the results reported previously by Hu et al. [36] although the pattern of dynamic changes has a difference. Data from multiple reaction monitoring profiling found that the number of carbons and unsaturation of fatty acyl residues decreased in both triglycerides (TGs) and phosphatidylglycerols (PGs) with the increase in lactation days from 0 to 14 [37]. The modules established from our data apparently were consistent with the profiling, indicating that the changes during lactation might be associated with the milk FA composition in TGs and PGs. Thus, changes in our dynamic module are consistent with enzyme kinetic effects. As pointed out by Suarez-Trujillo et al. [37], it might reflect biological and metabolic activity of liver and mammary gland of sows, and the development needs of the neonatal pigs.

Maternal clofibrate increased FA oxidation measured on d 1 and the increase was higher in pigs from sows fed 0.5% versus 0.25% clofibrate, reflecting a dose response and placental transfer efficiency. Similar results were obtained from our previous studies in sows receiving a diet with 0.8% clofibrate (w/w) from d 105 to 113 of gestation [9], demonstrating again that maternal clofibrate can be transmitted to fetus through the placenta and induce FA oxidation in the newborn. The induction assumed to be due to an increase in CPT I and ACO activities observed in previous study [19]. However, we noticed that the increased FA metabolism measured in 1-day-old pigs tended to be attenuated with the increase in postnatal age from d 1 to 7 of lactation. Indeed, a significant increase in abundance of *CPT I* and *ACO* was not detected in the case of promoted expression in *PPARα* and *RXRα*. The FA oxidation induced by maternal clofibrate supplementation during gestation period reduced with postnatal time [9]. The differences in FA oxidative metabolism between control and clofibrate treatment were not measurable after d 7, supporting that the maternal clofibrate might not be transferred to newborn pigs via milk, or the milk transfer could be minimum (negligible), in which the capability of clofibrate uptake by mammary tissues needs to be investigated. Interestingly, feeding high level of clofibrate increased ESP production as compared to control on d 1, 7 and 19, causing the total metabolites was also higher in high-level clofibrate than control on those days. Because PPARα stimulates FA transport protein and acyl-CoA synthetase expression and increases hepatic FA uptake and esterification [38], whether the increased ESP after d 7 was associated with an increased FA transport protein and acyl-CoA synthesis is not known. Especially the responses of genes associated with de novo FA synthesis in offspring to maternal clofibrate administration were not measured in this study. Determining the protein and enzyme expression and identifying the ESP products as well as the persistent impacts of maternal clofibrate administration are needed in future studies.

Although clofibrate feeding increased FA oxidation on d 1 and esterification measured on d 7 and 19, the increase did not change the distribution between CO_2_, ASP and ESP. The results stressed the primary role of CPT I in increasing FA metabolism induced by maternal clofibrate. However, the FA transferred into cytoplasm apparently preferred to be esterified with the increase of postnatal age. This was consistent with the results obtained from studies in isolated hepatocytes [6], enterocytes [39] and adipose tissue [40].

Inhibition of TCA cycle via malonate, the inhibitor of succinate dehydrogenase decreased CO_2_ production, had no effect on ASP production. This metabolic fate was not changed with the postnatal age. The results were consistent with the findings observed in earlier studies [6, 7]. Because piglets have a limited ketogenic capacity at birth, inhibition of TCA cycle would not increase ketone bodies production and induce changes in ASP production. Results from the current study implied that the ketogenic capacity was not significantly improved during the suckling period. Apparently, the failure to increase ketone body production when inhibiting TCA cycle is associated with the inability to increase mitochondrial HMGCS and acetoacetate-CoA deacylase activity [41] although clofibrate could stimulate the gene expression. Moreover, we found that the abundance of 3-hydroxy-3-methylglutaryl-CoA lyase (HMGCL) was reduced in pigs from sows with clofibrate administration. This finding is particularly important for further understanding of the regulation of ketogenic activity because HMGCL, located in both the mitochondrial matrix and the peroxisomes, catalyzes the cleavage of HMG-CoA to acetoacetic acid and acetyl-CoA, the last step of ketogenesis [42]*.* However, with the increase in postnatal age, the ASP decreased, and ESP increased. The increase in ESP was greater in treatment with malonate than without malonate in 4- and 19-day-old piglets, suggesting that the inhibiting TCA cycle activity could feedback inhibit FA oxidation. Addition of carnitine in the homogenate significantly increased both CO_2_ and ASP production. The stimulation of CO_2_ production was higher in d 1 than all other ages, while the increase in ASP was higher on d 7, 14 and 19 than d 1, suggesting that the hepatic carnitine amount from the storage, milk uptake and endogenous synthesis was not sufficient for optimizing FA metabolism rate during suckling period. Similar conclusions were obtained also via supplementation of carnitine to liver homogenate from 6-day-old pigs fed with milk formula [16]. Moreover, supplementation of L-carnitine to sows during gestation and lactation increases carnitine concentration in the milk and promoted piglets’ growth rate [43, 44], supporting our conclusion from this study. Further, the addition had no effect on ESP production. Adding carnitine with malonate together had a similar response to CO_2_ production, but significantly increased the ASP production. These results indicated that carnitine, as a substrate of the enzymes for converting CoAs to carnitines, promoted FA transfer from cytoplasm to mitochondria/peroxisome and FA oxidation in mitochondria/peroxisome with no effect on the inhibition of TCA activity. A similar amount of ASP obtained from the carnitine treated incubations regardless of whether there was malonate or not, suggesting that the acetyl-CoA generated from β-oxidation might transform to acetyl-carnitine to maintain the FA β-oxidation rate. This is particularly beneficial for fat metabolism in neonatal piglets with low ketogenic capacity and potential limitation in TCA activity.

Interestingly, adding carnitine had no impact on the percentage of CO_2_ production, but increased the percentage of ASP production and decreased ESP percentage. This demonstrated that the effect of carnitine on FA metabolism was completed through modification of the activity of the enzymes associated with the transfer of FA from CoAs to carnitines for β-oxidation or acetyl-CoA generated from the β-oxidation, subsequently increasing ASP production. Inhibition of the TCA cycle decreased the percentage of CO_2_ and had no impact on the percentage of ASP with or without adding carnitine. However, the inhibition increased the percentage of ESP on d 7 and 14 as compared to control, suggesting that the metabolic modification induced by malonate inhibition is related to the postnatal age.

## Conclusion

Maternal supplementation of clofibrate during late gestation and early lactation increases FA oxidative metabolism at birth and improves growth performance in the first week after birth. However, the impacts of maternal clofibrate were not observed in the piglets after 7 days of age. No clofibrate or clofibrate metabolites were detected in sow milk collected during and after 7 lactation days. These results imply that maternal clofibrate was not transferable via milk to suckling piglets or the transfer is negligible at the dose of 0.5% in the maternal diet. Hepatic FA oxidation decreases, and esterification increases linearly with increasing postnatal age. Carnitine availability is critical for neonatal pigs to maintain a high FA oxidation rate during the suckling period.

## Supplementary Information

**Additional file 1.** Primer sequences.

**Additional file 2.** Changes in milk fatty acids during suckling period. Data are least squares means (*n* = 9) ± SEM (standard error of the mean) following the quadratic changes (*P* < 0.0001). **A** Saturated fatty acids; **B** Monounsaturated fatty acids; **C** Polyunsaturated fatty acids; **D** Total saturated fatty acid and polyunsaturated fatty acids. Solid symbols indicate measured concentrations (% of the total identified fatty acids) and lines (solid and dash lines) indicate predicted concentrations (%).

## Data Availability

All data generated during this study are available from the corresponding authors on reasonable request.

## Abbreviations

ASP: Acid soluble products
ESP: Esterification products
FA: Fatty acid
MUFA: Monounsaturated fatty acid
PGs: Phosphatidylglycerols
PPARα: Peroxisome proliferator-activated receptor-alpha
PUFA: Polyunsaturated fatty acid
SFA: Saturated fatty acid
TCA: Tricarboxylic acid
TGs: Triglycerides
