# Supplementary material for: Effects of maternal feeding of clofibrate on hepatic fatty acid metabolism in suckling piglet
Source: J Anim Sci Biotechnol. 2024 Dec 5;15:163. doi: 10.1186/s40104-024-01104-6 (PMC11619605; doi:10.1186/s40104-024-01104-6)
Supplement: Supplementary file 1 — Additional file 1. Primer sequences. [file 40104_2024_1104_MOESM1_ESM.docx]

**Additional file 1** Primer sequences

| **Gene** | **Forward primer(5'→3'), Sen** | **Reverse primer(5'→3'), Anti** | **Amplicon size, bp** | **NCBI (Gene Bank)** |
| --- | --- | --- | --- | --- |
| *CPTIα* | *TCA CAA GCG AAT TTG AGT GC* | *AAA TTC AGA CCG CAG TTT CG* | *242* | *AF288789; sus* |
| *ACOX1* | *GGT CCA TCC ACG CTG TCT TA* | *CAC GTG GGT GAC TTG AGA CT* | *119* | *NM_001101028.1; sus* |
| *HMGCS* | *AAA TCC TTG GCT CGC CTG AT* | *GCG TAG GTG TCT TCC AGC TT* | *200* | *NM_214380.2; sus* |
| *RXRα* | *CTC CAT AGC CGT GAA GGA CG* | *CTG CTC GGG GTA CTT GTG TT* | *286* | *XM_021071446.1; sus* |
| *CAT* | *GAG CCT ACG TCC TGA GTC TC* | *TTG ATG CCC TGG TCA GTC TT* | *171* | *NM_214301.2; sus* |
| *ABCD3* | *TCT GGG GCC AAC GTT CTA AT* | *TCG AAG TGT TCC AAG GGT CA* | *168* | *NM_001244133.1; sus* |
| *PPARα* | *GCT GGA CGA CAG TGA CCT TT* | *AGC ACA TGC ACG ATA CCC TC* | *173* | *NM_001044526.1; sus* |
| *MYLCD* | *GAT CTC CAG CAC CAT CCA GA* | *ACC ACC CGC TTT ATC AGG AA* | *159* | *XM_021093740.1; sus* |
| *CYP4* | *TGT ACA ACC TGG CAA AGC AC* | *GGT ACA GCG ATG GGA GAT GA* | *188* | *XM_003123433.3; sus* |
| *ACO* | *CTC GCA GAC CCA GAT GAA AT* | *TCC AAG CCT CGA AGA TGA GT* | *218* | *AF185048; sus* |
| *CS* | *GGA AGT GCT TGT TTG GCT GA* | *GGC AGG TGT TTC AGA GCA AA* | *189* | *NM_214276.1; sus* |
| *HMGCL* | *AGG AAG TGG CGG TCT TTG TA* | *AGA TCT CGT AGC AGC CCA TC* | *237* | *XM_003356200.4; sus* |
| *SLC25a20* | *TGG GTT TGG ATT GGG GAA GA* | *CGA ATT CCA GAC TCC CGG TA* | *225* | *XM_003483178.4; sus* |
| *MCAD* (*ACADM*) | *GGC CAA CGA TGT TCA GAT ACA A* | *GGT ATT TCG GCG ACC AGA ATC* | *333* | *NM_214039.1; sus* |
| *LCAD* (*ACADL*) | *TTG GAG GGG ACT TGT ACT CG* | *CCA TCC TTC TTG GCA TTT GT* | *247* | *NM_213897.1; sus* |

*CPTIα* Carnitine palmitoyltransferase I alpha, *ACOX1* Peroxisomal acyl-CoA oxidase 1 isoform 1, *HMGCS* 3-Hydroxy-3-methylglutaryl-CoA synthase, *RXRα* Retinoid X receptor alpha, *CAT* Catalase, *ABCD3* ATP Binding cassette subfamily D member 3, *PPARα* Peroxisome proliferator-activated receptor alpha, *MYLCD* Malonyl-CoA decarboxylase, *ACADM* Acyl-CoA dehydrogenase medium chain, *CPY4* The cytochrome P450 4, *ACO* Acyl-CoA oxidase, *CS* Citrate synthase, *ACADL* Acyl-CoA dehydrogenase long chain, *HMGCL* 3-Hydroxy-3-methylglutaryl-CoA lyase, *SLC25a20* Solute carrier family 25 member 20
