# Supplementary material for: Effects of maternal feeding of clofibrate on hepatic fatty acid metabolism in suckling piglet
Source: J Anim Sci Biotechnol. 2024 Dec 5;15:163. doi: 10.1186/s40104-024-01104-6 (PMC11619605; doi:10.1186/s40104-024-01104-6)
Supplement: Supplementary file 2 — Additional file 2. Changes in milk fatty acids during suckling period. Data are least squares means (n = 9) ± SEM (standard error of the mean) following the quadratic changes (P < 0.0001). A Saturated fatty acids; B Monounsaturated fatty acids; C Polyunsaturated fatty acids; D Total saturated fatty acid and polyunsaturated fatty acids. Solid symbols indicate measured concentrations (% of the total identified fatty acids) and lines (solid and dash lines) indicate predicted concentrations (%). [file 40104_2024_1104_MOESM2_ESM.pdf]

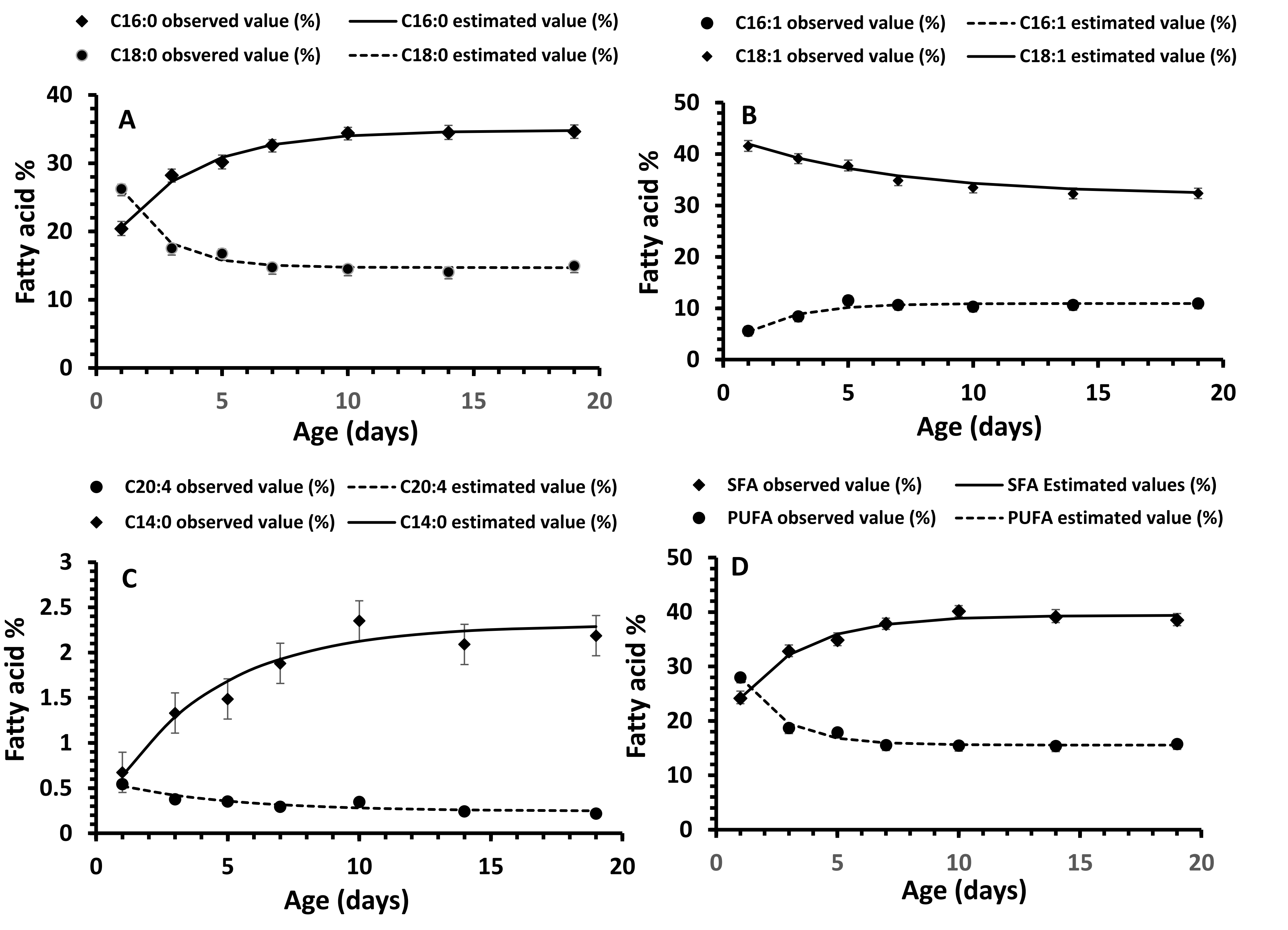
C16:0 observed value (%)
C16:0 estimated value (%)
C18:0 obsvered value (%)
C18:0 estimated value (%)
A
Fatty acid %
Age (days)
C16:1 observed value (%)
C16:1 estimated value (%)
C18:1 observed value (%)
C18:1 estimated value (%)
B
Fatty acid %
Age (days)
C20:4 observed value (%)
C20:4 estimated value (%)
C14:0 observed value (%)
C14:0 estimated value (%)
C
Fatty acid %
Age (days)
SFA observed value (%)
SFA Estimated values (%)
PUFA observed value (%)
PUFA estimated value (%)
D
Fatty acid %
Age (days)
